# Pharmacokinetics/pharmacodynamics of marbofloxacin in a *Pasteurella multocida* serious murine lung infection model

**DOI:** 10.1186/s12917-015-0608-1

**Published:** 2015-12-02

**Authors:** Ying Qu, Zhenzhen Qiu, Changfu Cao, Yan Lu, Meizhen Sun, Chaoping Liang, Zhenling Zeng

**Affiliations:** National Reference Laboratory of Veterinary Drug Residues (SCAU), College of Veterinary Medicine, South China Agriculture University, Guangzhou, China

**Keywords:** Marbofloxacin, Pharmacokinetics/Pharmacodynamics, *Pasteurella multocida*, Mouse-lung model

## Abstract

**Background:**

Marbofloxacin is a third-generation fluoroquinolone developed solely for veterinary medicine with a broad spectrum of antibacterial activity against some Gram-positive and most Gram-negative bacteria, including the bovine respiratory tract pathogen, *Pasteurella multocida*. The objective of our study was to elucidate the pharmacokinetics and pharmacodynamics of marbofloxacin in a *Pasteurella multocida* infected murine lung model, and to estimate the magnitudes of pharmacokinetics-pharmacodynamics parameters associated with various effects.

**Results:**

The pharmacokinetic studies revealed marbofloxacin kinetics in infected mice were linear over a dose ranging from 1.25 to 10 mg/kg of body weight. The protein binding in the plasma of neutropenic infected mice was 29.77 %. The magnitudes of the ratio of the free-drug area under the concentration-time curve over 24 h to MIC (*f*AUC_0-24h_/MIC) associated with static effect, a 2 log_10_ reduction and a 3 log_10_ reduction of bacterial counts were 40.84, 139.34, and 278.08 h, respectively.

**Conclusions:**

Based on the dose range study, marbofloxacin exhibited concentration-dependent killing and the *f*AUC/MIC was the PK/PD index that correlated best with efficacy (R^2^ = 83 %). On the basis of a bactericidal effect goal of *f*AUC_0-24h_/MIC of 278.08 h, if marbofloxacin is used for the treatment of *P. multocida* serious lung infection with an MIC_90_ of 0.12 μg/ml, the current dose (2 mg/kg) would fail to achieve a bactericidal effect. It would benefit from higher doses (4 ~ 6 mg/kg) than those commonly used in clinical practice.

**Electronic supplementary material:**

The online version of this article (doi:10.1186/s12917-015-0608-1) contains supplementary material, which is available to authorized users.

## Background

*Pasteurella multocida* is a pathogenic Gram-negative bacterium that is a common inhabitant of the upper respiratory tract of calves [1]. It may cause a wide spectrum of diseases ranging from septicemia to pneumonia [2]. Marbofloxacin, a synthetic third-generation fluoroquinolone has been developed solely for veterinary use and displays a broad spectrum of antimicrobial activity against most Gram-negative and some Gram-positive bacteria [3–5]. It has been approved for the treatment of respiratory, urinary tract, soft tissue and dermatological infections in cattle, pigs, dogs, and cats [6–8].

Using pharmacokinetics and pharmacodynamics (PK-PD) principles is a widely accepted approach for optimization of antimicrobial therapy [9, 10]. Three principal PK-PD surrogate markers of efficacy (AUC_0→24h_/MIC, C_max_/MIC, T_>MIC_) have been used as a basis for dose optimization by quantifying the potency and efficacy of antimicrobials against target pathogens [11, 12].

Previous studies have demonstrated that marbofloxacin displays a concentration-dependent killing mechanism of action and the AUC_24h_/MIC surrogate marker is the PK-PD parameter best linked with efficacy [3, 13, 14]. There are numerous studies describing the characteristics of the time-kill profiles and growth inhibition of marbofloxacin against *P. multocida*. However, the pharmacodynamic properties of marbofloxacin were elucidated by using in vitro dynamic models or ex vivo using calf serum, exudate, and transudate harvested from a tissue cage model. The results were established by modelling in vitro time-kill data with pharmacokinetic properties of marbofloxacin. The goal of our studies was to characterize the in vivo activity of marbofloxacin in a mouse model of serious *P. multocida* infection, and to determine the magnitude of PK/PD parameter predictive of efficacy. Today, animal pharmacokinetic-pharmacodynamic (PK-PD) infection models serve as a cornerstone of the preclinical assessment process for antibacterial agents. The magnitude of exposure identified for bacterial stasis in immunocompromised animals was similar to the exposure threshold associated with good clinical outcomes for patients treated with oritavancin or linezolid for bacteremia [15]. A neutropenic mouse model is always used in human medicine research [16]. We can exclude the host defense and only analyze the effects of drug on bacteria with this model, which is known to be relevant for drawing conclusions about drug PK/PD with humans [16, 17].

## Methods

### Bacteria and antibiotic

*P. multocida* strain *CVCC 411*, isolated from a buffalo that died from hemorrhagic septicemia, was obtained from National Veterinary Microorganism Strains Preservation Management Centre (Beijing, China). Marbofloxacin was a 10 % injectable aqueous solution obtained from Veterinary Pharmaceutical Corporation (Yuanzhen Co., Ltd, Hebei, China). Marbofloxacin reference standard was purchased from Dr. Ehenstorfer GmbH company (Germany) and ofloxacin was aquired from the National Institute for Food and Drug Control (Beijing, China).

### In vitro susceptibility testing

The MIC of marbofloxcin was determined by broth microdilution methodology according to the Clinical and Laboratory Standards Institute (CLSI) recommended methods and quality control requirements. Susceptibility testing was performed in triplicates.

### Inoculum preparation

A few colonies of freshly grown *P. multocida CVCC 411* from Tryptic Soy agar (Guangdong Huankai Microbial Sci. & Tech. Co., Ltd., Guangzhou, China) plates supplemented with 5 % sheep blood (Puboxin Biotechnology Co., Ltd., Beijing, China) were cultured in Mueller–Hinton II broth (Becton Dickinson, Sparks, MD, USA) with shaking at 200 rpm at 37 °C for 10 h (final cell count approximately 10^9^ CFU/mL). Bacteria were collected by centrifugation at 3,000 g for 10 min and re-suspended in 0.9 % NaCl to obtain a final suspension containing 10^10^ CFU/mL. This suspension was then diluted 100 times to obtain a bacterial concentration at 10^8^ CFU/mL.

### Animals

Six-week-old, specific-pathogen-free, female ICR mice (Medical Experimental Animal Center of Guangdong Province, Guangzhou, China) weighing between 22 and 24 g were used for the experiments. All animal studies were approved by the Animal Research Committees of South China Agriculture University. The animals were maintained in accordance with the American Association for Accreditation of Laboratory Animal Care criteria. All sections of this report adhere to the ARRIVE Guidelines for reporting animal research. A completed ARRIVE guidelines checklist is included in Additional file 1.

### Neutropenic mouse lung infection model

Before the mice were infected with *P.multocida* using endotracheal intubation, the mice were rendered neutropenic by injecting cyclophosphamide (Aladdin Co., Ltd., Shanghai, China) intraperitoneally 4 days (150 mg/kg of body weight) and 1 day (100 mg/kg of body weight) [18]. On the 6th day, the mice were anesthetized by injecting 1 % pentobarbital sodium (XiangBo Biotechnology Co., Ltd., Guangdong, China) solution (50 mg/kg) intraperitoneally. A simple 22G “Y” type intravenous catheter (PUYI Medical Devices Co., Ltd., Shanghai, China) that consisted of an IV catheter connected to a PVC flexible pipe was used to intubate the mice. A 50 μL saline suspension of *P. multocida CVCC411* containing 10^8^ CFU/mL described above was injected into the PVC flexible pipe and then kept horizontal. The anesthetized mouse was placed on an adjustable-angled support suspended by its upper incisors; the tongue was gently pulled out of the mouth. Using a light source illuminating the neck of the mouse, we can clearly observe the glottis opening and then closing alternately. The catheter was inserted through the glottis and if the catheter was successfully inserted into the trachea, the inoculum in the PVC flexible pipe would fluctuate following the breath of the mouse. We used 0.8 mL gas to thrust the 50 μL bacterial inoculum into the lung (this process operated approximately for 2 s), thereafter, mouse was held in a vertical position for 15 s. Antimicrobial therapy was initiated 10 h when the mice showed lethargy and dyspnea after the infection of *P. multocida.*

### PK studies

The neutropenic mice were infected with *P. multocida* as described above, and 10 h later were administered with single subcutaneous doses of 1.25, 2.5, 5, or 10 mg/kg marbofloxacin in 0.2 mL volumes. Groups of four mice were each sampled by retro-orbital puncture at 0.083, 0.25, 0.5, 0.75, 1, 2, 4, 6, 8, 12 and 24 h after dosing. The total volume collected from each individual mouse was less than 10 % of the total blood volume, namely each mouse was sampled about three times and each time a blood sample of approximately 0.25 mL was obtained. Blood samples were centrifuged at 7,000 g for 10 min at 4 °C, the plasma were removed and stored at −20 °C until the assay. Marbofloxacin concentrations were determined using a HPLC method with fluorescence detection (excitation and emission wavelengths were 295 nm and 500 nm). Briefly, marbofloxacin was extracted as follows: 0.1 mL plasma, spiked with 10 μL internal standard-ofloxacin, was added to 3 mL of trichloromethane and vortexed for 30s. The organic layer was collected after centrifugation for 10 min at 3,000 g, and then concentrated to dryness under a stream of nitrogen. A 0.2 mL mobile phase was used to re-suspend the dried sample and a 20 μL aliquot was taken and injected into HPLC for analysis. The standard calibration curve of marbofloxacin was linear for concentrations ranging from 0.01 to 5 μg/mL. The intra-day and inter-day precision levels varied from 4.13 % to 7.18 % and from 6.70 % to 8.24 %, respectively. The protein binding in the plasma of neutropenic infected mice was determined by ultrafiltration methods. The level of binding was measured with marbofloxacin concentrations of 0.05, 0.5 and 5 μg/mL.

### PD parameter determination

The neutropenic mice were infected with *P. multocida* using endotracheal intubation as previously described; we used twenty control mice which were not treated to assess the growth of *P. multocida* in the lungs. Groups of five mice were sacrificed 2, 5, 10 and 24 h after the inoculation and the amount of bacteria in the lungs was counted. The neutropenic mice were infected with *P. multocida* 10 h prior to the start of therapy. Groups of five mice were treated for 24 h with single marbofloxacin doses from 0.625 to 10 mg/kg/24 h (0.625, 1, 1.25, 2, 2.5, 4, 5, 8, 10 mg/kg). The drug was administered subcutaneously in 0.2 ml volumes. The treated mice were humanely killed 24 h after initiation of treatment; the lungs were aseptically removed and homogenized in 5 mL of sterilized saline, individually. Bacterial counts of each lung were determined by serial 10-fold dilutions of homogenates, 20 μL drops of the successive dilutions were dropped in triplicate on a TSA (supplemented with 5 % sheep blood) surface and waited to dry before incubating at 37 °C. If the bacterial counts were less than 250 CFU/lung, 100 μL of the homogenates were plated in triplicate on agar. The lowest level of detection was 50 CFU/lung (equivalent to one colony per plate). The untreated control mice were humanely killed just before treatment and after 24 h.

### PK and PK/PD analysis

The data obtained with a single subcutaneous injection of 1.25, 2.5, 5 and 10 mg/kg marbofloxacin were analyzed separately using WinNonlin version 5.2 (Pharsight Corporation). Marbofloxacin concentration-time data were best described using the noncompartment model with extravascular input. According to dose proportionality from the obtained PK parameters, we used them to estimate the AUC_0-24h_, C_max_ for each tested mabofloxacin dosing for which no kinetics were determined. The PK/PD analysis was performed by using the inhibitory effect E_max_ model. This model is described by the following equation: E = E_max_-(E_max_-E_0_)*(C/(EC_50_ + C)), where E is the change in log_10_CFU/lung after 24 h in treated mice compared to the initial log_10_CFU/lung in untreated control mice; E_max_ is the log change in CFU per lung, comparing 0 and 24 h in the untreated control mice; E_0_ is the change in log_10_CFU/lung between the treated mice with untreated mice after the 24 h period of the study, when the detection limit is reached; C is the PK/PD parameter (e.g. *f*AUC/MIC, *f*Cmax/MIC, *f*T_>MIC_); EC_50_ is the C value at which 50 % of the maximal antibacterial effect is produced. These PD parameters were calculated using the nonlinear regression program (WinNonlin, Pharsight Corporation).

## Results

### In vitro susceptibility testing

The MIC of marbofloxacin for *P. multocia CVCC 411* was 0.031 μg/mL by the broth microdilution methodology.

### Pharmacokinetic study

**Fig. 1:**
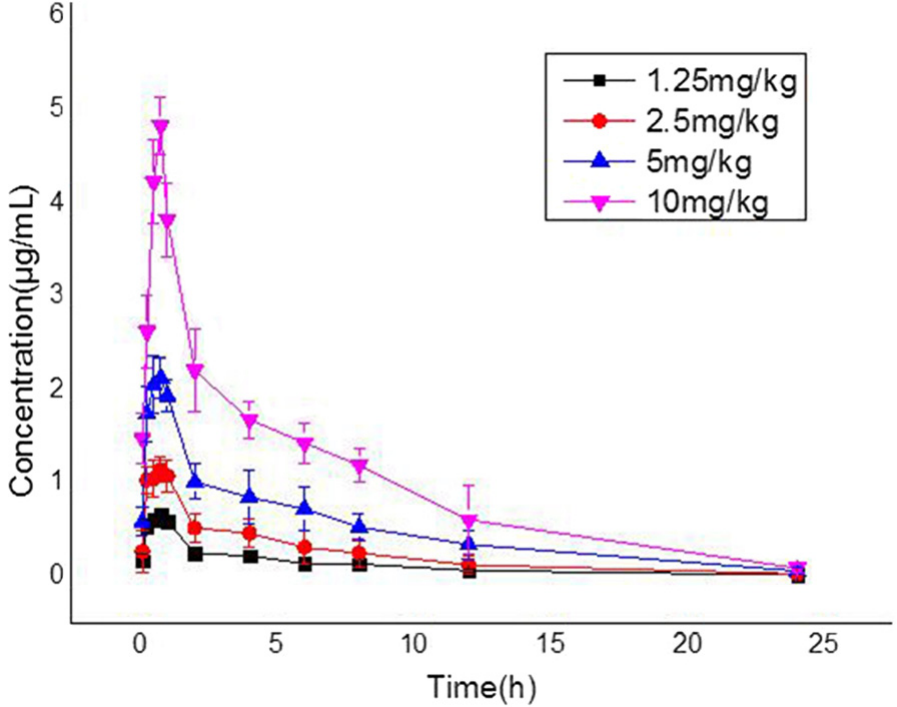
Pharmacokinetic profiles of marbofloxacin in neutropenic mice. The neutropenic mice were infected with *P. multocida* as described above, and 10 h later administered with single subcutaneous doses of 1.25, 2.5, 5, or 10 mg/kg marbofloxacin in 0.2 mL volumes. Groups of four mice were each sampled by retro-orbital puncture at 0.083, 0.25, 0.5, 0.75, 1, 2, 4, 6, 8, 12, 24 h after dosing. Marbofloxacin concentrations were determined using a HPLC method with fluorescence detection. Each symbol represents the mean ± standard deviation of the levels in the sera of three mice

The time-concentration curves of marbofloxacin in the plasma of infected neutropenic mice after single subcutaneous doses of 1.25, 2.5, 5, and 10 mg/kg are shown in Fig. 1. The PK data obtained with different marbofloxacin doses were analyzed using a noncompartment model with extravascular input, and the obtained values of marbofloxacin pharmacokinetic parameters are shown in Table 1. The PK parameters were dose-dependent, where the AUC/dose values for the escalating single doses ranged from 2.29 to 2.41, and the C_max_/dose values ranged from 0.43 to 0.53. The plasma protein binding of marbofloxacin at concentrations of 0.05, 0.5, 5 μg/ml were 33.04 ± 2.37 %, 25.70 ± 1.18 %, 30.55 ± 0.10 %. And the mean value of 29.77 % of the plasma protein binding was used to calculate the free plasma concentrations. Thus, we can obtain the free PK parameter values.

**Table 1:** Pharmacokinetic parameters obtained from plasma concentrations after administration of marbofloxacin in infected neutropenic mice

Marbofloxacin dose (mg/kg)	C_max_ mean ± SD (μg/ml)	T_max_ (h)	AUC (μg.h/ml)	Cl (ml kg^−1^ h^−1^)	T_1/2_ (h)
1.25	0.66 ± 0.07	0.75	2.92	411.2	5.40
2.5	1.14 ± 0.14	0.75	5.73	420.3	5.23
5	2.13 ± 0.22	0.75	12.05	400.9	5.00
10	4.83 ± 0.30	0.75	23.90	408.9	4.29

C_max_: maximum concentration of drug in plasma

T_max_: time to maximum concentration of drug in plasma

AUC: area under the concentration-time curve

Cl: clearance

T_1/2_: half-life

### Lung infection model

The intubation method in this study was based on other endotracheal intubation methods [19] and improved by using a 22G “Y” type intravenous catheter. This method features well in terms of repeatability, and it is non-invasive, inexpensive and rapid. About 6.81 log_10_CFU/mouse was inoculated via the trachea, but by two hours after the inoculation, the bacterial load in mice was 6.15 ± 0.21 log_10_ CFU/lung. Five hours after inoculation the bacterial burden was 6.60 ± 0.15 log_10_ CFU/lung and the mice had no clinical signs of infection. Ten hours after the inoculation, the mice were lethargic with dyspnea and the bacterial population reached 7.38 ± 0.19 log_10_ CFU/lung, and by 24 h after inoculation, the bacterial population increased to 8.82 ± 0.16 log_10_ CFU/lung, and the mice were on the verge of death.

### PK/PD analysis

**Fig. 2:**
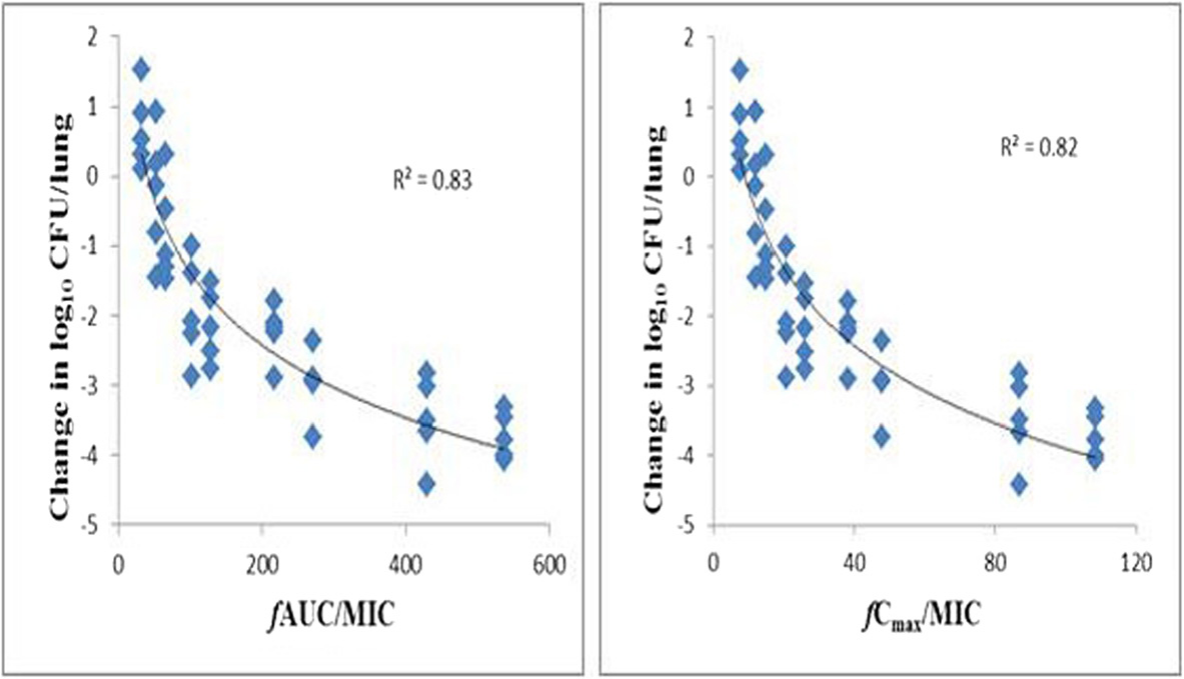
Relationships between marbofloxacin’s *f*AUC_24_/MIC, *f*C_max_/MIC and microbiological efficacy for *pasteurella multocida*. The neutropenic mice were infected with *P. multocida* 10 h prior to the start of therapy. Groups of five mice were treated for 24 h with single marbofloxacin doses from 0.625 to 10 mg/kg/24 h (0.625, 1, 1.25, 2, 2.5, 4, 5, 8, 10 mg/kg). After 24 h of therapy, the amount of bacteria in the lungs was counted. The figure above reveals the relationships of marbofloxacin’s *f*AUC_24_/MIC and *f*C_max_/MIC for *pasteurella multocida CVCC 411* with the change in the log_10_ number of CFU/lung after 24 h of therapy. Each symbol represents a single lung tissue *per* mouse. R^2^ is the correlation coefficient

(i) Total bacterial populations. Total bacterial populations after 24 h of marbofloxacin treatment are reported in Table 2. At the start of therapy, mice were infected with 7.12 ± 0.12 log_10_ CFU/lung of *P. multocia CVCC 411*. The untreated control mice died in around 24 h post-challenge and the organisms grew 1.95 ± 0.19 log_10_ CFU/lung. The relationships between efficacy and each of the PK/PD indices (*f*AUC_0-24h_/MIC, *f*Cmax/MIC) are shown in Fig. 2. *f*AUC_0-24h_/MIC correlated well with microbiological efficacy (R^2^ = 0.83), and a similar relationship was observed for the *f*Cmax/MIC correlated with efficacy (R^2^ = 0.82). (ii) PK/PD parameters determining efficacy: The dose–response relationships with single doses of marbofloxacin for *P. multocia* were evaluated using inhibitory Effect E_max_ model. The PK/PD model parameter estimates for the *f*AUC_0-24h_/MIC index and the values of *f*AUC_0-24h_/MIC required for static effect, 2 and 3 log_10_ reduction of bacterial burden are shown in Table 3. The highest dose of 10 mg/kg marbofloxacin, corresponding to *f*AUC_0-24h_/MIC values of 537.12 h, reduced the bacterial burden by more than 3 log. The *f*AUC_0-24h_/MIC values of 91.08 ± 16.49 h, equivalent to 1.77 ± 0.32 mg/kg for marbofloxacin, performed 50 % of the maximum antibacterial effect.

**Table 2:** Total bacterial populations after 24-h exposure to different doses of marbofloxacin

Treatment group and time point	Total dose (mg/kg)	Bacterial population (mean ± SD) (log_10_ CFU/lung)
Untreated controls	0	
At start of treatment^a^		7.12 ± 0.12
24 h after inoculation^b^		9.07 ± 0.19
24 h after start of treatment		**
Single administration	0.625	7.79 ± 0.56
	1	6.87 ± 0.91
	1.25	6.31 ± 0.74
	2	5.20 ± 0.74
	2.5	4.97 ± 0.52
	4	4.88 ± 0.41
	5	4.14 ± 0.49
	8	3.64 ± 0.63
	10	3.40 ± 0.32

^a^Total bacterial population 10 h after the time of infection

^b^24 h after the inoculation was the time that the mice approached to death, and the lungs were removed for calculation the bacterial population

**The mice were already dead

**Table 3:** The PK/PD parameter estimates for the *f*AUC/MIC index and the *f*AUC/MIC required for various antibacterial effects

Parameter	Unit	Mean ± SD
E_max_	log_10_CFU/lung	2.08 ± 0.26
E_0_	log_10_CFU/lung	−4.67 ± 0.32
*f*AUC/MIC for EC_50_	h	91.08 ± 16.49
*f*AUC/MIC for static effect	h	40.84
*f*AUC/MIC for 1 log_10_ kill	h	76.88
*f*AUC/MIC for 2 log_10_ kill	h	139.34
*f*AUC/MIC for 3 log_10_ kill	h	278.08

## Discussion

In the current study, we used a neutropenic mouse-lung model to characterize the PD profiles of marbofloxacin, a fluoroquinolone of veterinary interest. We infected the mice with *P. multocida*, which eventually led to the development of septicemia, This murine lung infection model was also used to elucidate the magnitude of the *f*AUC_0-24h_/MIC index required for various effects.

Previous in vitro and in vivo studies demonstrated that inoculum size influenced the pharmacodynamics of antimicrobial drugs. Indeed, the different inoculum sizes were directly inoculated into broth and animals but the natural growth of the bacterial was not taken into account in these studies [20, 21]. In the present study, when the mice had slight clinical signs, we administered marbofloxacin at this time to mimic “conventional” curative treatment. In an earlier study, Ferran et al. [22] referred to the later “conventional” curative treatment in a mouse-lung model of *P. multocida* infection studies.

Previous in vivo studies had shown that bacterial infections altered the PK of drugs, including fluoroquinolone antimicrobials, such as marbofloxacin [16, 21, 23, 24]. We evaluated the PK of marbofloxacin in *P. multocida-*infected mice with single subcutaneous doses of 1.25, 2.5, 5, 10 mg/kg. The PK data in Table 2 showed dose-proportionality for marbofloxacin in *P.multocida* infected mice, with the AUC and C_max_ proportional to the dose. This dose-proportionality was also previously demonstrated in mice infected with *Escherichia coli* in the thighs [20]. In this study, the AUC value of marbofloxacin in mice receiving 10 mg/kg was 23.90 μg.h/ml, which was in accordance with the AUC values of the mice receiving 20 mg/kg marboflxacin for late administration after inoculation of *P. multocida* (54 μg.h/ml) [22]. The clearance of 410.33 ± 7.98 ml kg^−1^ h^−1^ of the four doses of marbofloxacin was similar to the clearance reported in the mice receiving 20 mg/kg marbofloxacin for late administration after inoculation of *P. multocida* (370 ml kg^−1^ h^−1^) [22]. The evolution of the disease induced a lower clearance of marbofloxacin, as previously observed [20]. The elimination half-life was 4.98 ± 0.49 h for the four doses in infected mice, and the value was higher in this study than the previously reported values. The production of endotoxins by gram-negative bacterial was shown to decrease the activity of hepatic metabolism, the renal blood flow, and the glomerular filtration rate, and might be the explanation for the alteration of the drug elimination [20]. Both organisms are gram-negative so endotoxins could have played a role in both infections. Since single-injection cures are of major importance to the cattle industry, we only administered a single treatment within 24 h in this study, rather than the multiple dosing regimens generally employed in other in vivo studies.

Several animal infection models have identified that the fluoroquinolones display the characteristics of concentration-dependent killing and the AUC/MIC ratio is the PK/PD index most predictive of the antibacterial effect [16, 25]. Based upon R^2^ values and visual examination of the fits, data from this lung infection model study confirmed that *f*AUC_0-24h_/MIC and *f*C_max_/MIC well correlated with efficacy of marbofloxacin against *P. multocida.* In the current investigation, the *f*AUC_0-24h_/MIC required to produce a static effect, 2 log_10_ reduction and 3 log_10_ reduction in bacterial counts against *P. multocida* were 40.84, 139.34, 278.08 h, respectively, which were higher than observed in previous studies. Potter et al. [14] found that the mean AUC_24h_/MIC values of marbofloxacin for no reduction, 3 log_10_ and 4 log_10_ reductions in bacterial count from the initial inoculum count, based on serum MICs, were 48.6, 64.9, and 74.8 h for *P. multocida*. Moreover, the corresponding values were 24.35, 44.24, and 64.36 h in the studies reported by Illambas et al. [13]. In this present study, the initial bacterial load (7.12 ± 0.12log_10_CFU/lung) was higher than a medium inoculum count of 5*10^6^ CFU/ml routinely used in time-kill studies. As previously observed, pathogen load impacts the drug concentration required for inhibition of growth, with a general expectation of a higher dosage requirement for higher pathogen loads [13]. Therefore, the higher initial *P. multocida* load in this murine lung infection model might explain the higher *f*AUC/MIC values for various magnitudes of effect. Previous reports found that for a low and a high inoculum of bacterial in the mouse infection model, a low and a high value of AUC_24_/MIC, respectively, were needed for bactericidal effect [20, 21]. These findings fully confirmed that the inoculum size influenced the values of pharmacokinetic/pharmacodynamic indices.

## Conclusions

The present study showed that *f*AUC/MIC and *f*C_max_/MIC well correlated with efficacy of marbofloxacin against *P. multocida*. The value of the 24 h static dose was a *f*AUC_0-24h_/MIC of 40.84 h and when the ratio reached 91.08 h, rates of microbiologic cure approached 50 %. Moreover, the results showed that a bactericidal action was not achieved until the *f*AUC_0-24h_/MIC had reached 278.08 h.

In conclusion, the antimicrobial PD in humans may be predicted from studies with animal models. According to the pharmacokinetic properties and the PK/PD parameters, the neutropenic mouse model has successfully predicted the dose of therapy in human medicine area [16, 17, 25]. Therefore, in this study, the recommended dose of marbofloxacin of 2 mg/kg for cattle against bovine respiratory disease produced a 24 h AUC of 10-15 μg.h/ml [5]. On the basis of a bactericidal effect goal of *f*AUC_0-24h_/MIC of 278.08 h, this model would predict that if marbofloxacin is used for the treatment of *P. multocida* serious lung infection with an MIC_90_ of 0.12 μg/ml, the current dose would fail to achieve a bactericidal effect. It would benefit from higher doses (4 ~ 6 mg/kg) than those commonly used in clinical practice. However, with drugs from the fluoroquinolone class, the presence of neutrophils can enhance antimicrobial activity by up to a factor of four to six [26]. Thus the above dosage was a theoretical value, which should further validate in clinical or field study.

## Additional file

Additional file 1:
**The ARRIVE Guidelines Checklist.** Animal Research: Reporting In Vivo Experiments. (PDF 391 kb)

## Abbreviations

*P. multocida*: *Pasteurella multocida*
MIC: minimum inhibitory concentration
PK: Pharmacokinetics
PD: Pharmacodynamics
AUC: area under the curve
C_max_: peak serum (or plasma) concentration
*f*AUC_24h_/MIC: the ratio of area under the free plasma/serum concentration-time curve over 24 h to MIC
*f*C_max_/MIC: the ratio of free peak serum (or plasma) concentration measured in vivo to MIC determined *in vitro*
CFU: Colony forming unit
CVCC: China veterinary culture collection
HPLC: high performance liquid chromatography
CLSI: clinical and laboratory standards institute
